# SERS-based detection of DNA methylation for cancer diagnosis: Cation-mediated adsorption to silver nanoparticles

**DOI:** 10.1371/journal.pone.0325539

**Published:** 2025-06-13

**Authors:** Stefania D. Iancu, Vlad Moisoiu, Bogdan Adrian Tigu, Andrei Stefancu, Oana M. Biro, Ciprian Tomuleasa, Nicolae Leopold

**Affiliations:** 1 Faculty of Physics, Babeș-Bolyai University, Kogalniceanu 1, Cluj-Napoca, Romania; 2 Department of Personalized Medicine and Rare Diseases, MEDFUTURE—Institute for Biomedical Research, Iuliu Hatieganu University of Medicine and Pharmacy, Louis Pasteur 4, Cluj-Napoca, Romania; 3 Department of Hematology, Ion Chiricuta Clinical Cancer Center, Cluj-Napoca, Romania; 4 Department of Hematology, Iuliu Hatieganu University of Medicine and Pharmacy, Cluj-Napoca, Romania; University of Baghdad, IRAQ

## Abstract

The high-throughput analysis of DNA methylation markers by label-free surface-enhanced Raman scattering (SERS) holds significant promise for advancing cancer detection. However, a deeper understanding of the factors governing DNA adsorption onto metal surfaces and the identification of reliable SERS bands indicative of DNA methylation levels are still needed. In this study, we evaluated the effects of several cations (Ca^2+^, Mg^2+^, Al^3+^, Be^2+^, Zn^2+^, Cu^2+^, Fe^2+^, and Na⁺) on the SERS signal of DNA and identified Ca^2+^ as providing the highest enhancement. Thus, the addition of 5x10^-4^ M Ca^2+^ yielded optimal SERS signals for genomic DNA extracted from calf thymus and from six human cell lines, including both benign and malignant types with varying methylation levels. Notably, the SERS activation effect due to Ca^2+^ could also be replicated by lowering the pH, suggesting that Ca^2+^ increases the signal enhancement by generating surface Ag⁺ which favors the adsorption of DNA. A strong positive correlation (R = 0.94, p = 0.005) was observed between the intensity of the SERS band at 790 cm^-1^ and the level of 5-methylcytosine, establishing this band as a robust marker for DNA methylation. This finding was further validated by monitoring methylation levels in a 180 bp DNA sequence from the promoter region of the SEPT9 gene, an FDA-approved biomarker for colorectal cancer. Additionally, the use of SYBR Green fluorescence assays revealed that hypermethylated genomic DNA exhibits greater affinity for silver surfaces compared to lower methylated DNA. Collectively, these findings provide important theoretical insights and practical directions for the development of future nanoparticle-based cancer detection assays utilizing methylation markers.

## Introduction

Epigenetic alterations, including clustered hypermethylation of CpG islands and global DNA hypomethylation, are believed to hold the potential to achieve the holy grail of modern oncology: multi-cancer early detection [1–4]. Typical methods for detecting DNA methylation, such as bisulfite-conversion based sequencing and PCR and methylation arrays, have significant drawbacks, as they are both costly and labour-intensive [1–4]. Surface-enhanced Raman scattering (SERS), a laser-based vibrational spectroscopy technique that employs plasmonic substrates, has been proposed as an alternative to overcome many of these limitations [5–13].

A major challenge in translational SERS detection is the insufficient understanding in two critical areas that we address in this study while investigating SERS for detecting cancer-associated changes in DNA methylation. The first relates to interfacial processes that govern SERS signal enhancement, particularly the roles of analyte adsorption and nanoparticle aggregation. The second concerns the choice between label-free and label-based strategies for implementing SERS as an analytical detection method. As we demonstrate, these two aspects are closely interconnected.

SERS substrates—typically colloidal silver or gold nanoparticles—enhance the Raman signal of molecules through interactions between the incident laser light and collective oscillations of conduction electrons within the metallic surface, known as localized surface plasmons [14] (for an in-depth discussion on this topic, see the excellent review by Garcia-Rico et al. [15] or our own previous review [8]).

A key experimental observation underlying the first critical area is that simply mixing an analyte with a SERS substrate often fails to produce a detectable signal. To “activate” the signal, the addition of salts such as NaCl, MgSO₄ or Ca(NO₃)₂ is typically required [8,16,17]. This salt-induced enhancement is commonly attributed to nanoparticle aggregation, which creates electromagnetic hotspots where analytes are physically trapped and the Raman signal is strongly amplified [18].

However, while aggregation remains a widely cited mechanism—even at relative low salt concentrations—our recent studies provide compelling evidence for an additional, critical role: cation-mediated analyte adsorption onto the metallic surface [19,20]. The most convincing support for this mechanism is the ability to sequentially detect multiple analytes coexisting in the same solution [8], an effect also demonstrated in the current study. This behavior cannot be fully explained by simple physical trapping in electromagnetic hotspots, highlighting the need to reassess the interplay between aggregation and surface chemistry in SERS signal generation.

A second debate concerns the strategy for implementing SERS for detecting biologically relevant analytes, including DNA from malignant cells. Many researchers have developed label-based methods that depend on specific chemical interactions between the analyte and SERS substrates functionalized with antibodies, nucleic acid probes, aptamers, and similar recognition elements [21]. The underlying idea is that the analytes binding to the functionalized SERS substrate would either alter the SERS signal of a reporter molecule or induce a shift in the plasmonic band of the substrate [14,21]. In contrast, we (and others) have advocated for the use of label-free SERS detection [17,19,20,22,23]. Our focus on this approach stems from our interpretation of experimental observations regarding the role of salts in SERS amplification: if the salt-induces the analyte adsorption and the subsequent SERS enhancement of the signal, this mechanism could, in principle, be tailored to achieve molecular specificity. Conversely, if amplification were driven by nanoparticle aggregation, one would not expect specificity. For label-free SERS strategies, the superior optical properties of silver nanoparticles are preferable to gold nanoparticles, the latter being more commonly used in label-based applications owing to the well-established gold surface chemistry and more uniform geometries [24].

On one hand, label-free SERS poses several challenges. Firstly, the interactions between various classes of analytes and the SERS substrate are often complex and difficult to control through chemical modifications such as changes in ionic strength or pH. Secondly, identifying appropriate SERS marker bands is frequently problematic—especially in complex biofluids such as plasma or urine—where band misassignments are common [8,25]. Similar difficulties are encountered in the analysis of DNA [9,26,27]. Finally, achieving specificity toward the target analytes remains a critical need. Identifying experimental configurations that meet these demands is often difficult; however, we believe that, in the long term, label-free approaches hold substantially greater promises for driving transformative advances.

DNA methylation serves as an ideal model system for developing label-free analytical methods, as it meets the aforementioned criteria. Thus, DNA is a polyanionic molecule [28], and its interaction with metal colloids is typically hindered by electrostatic repulsion from negatively charged surfactants (unless positive coating agents such as spermine are used [29]), creating a Coulomb barrier that can be selectively modulated through chemical means. Moreover, 5-methylcytosine, the hallmark modification of methylated DNA, exhibits distinct marker SERS bands [9,17]. Finally, since DNA methylation plays a crucial role in regulating gene expression by altering the conformation of the DNA double helix [30], this structural change expectedly influences how DNA interacts with proteins and other biomolecules [31–33], potentially also altering the interaction with SERS substrates [22,34,35]. Thus, methylation not only impacts cellular function but also modulates the physicochemical properties of DNA in ways that are accessible to spectroscopic interrogation.

In this study, we present compelling evidence supporting the specific detection of cancer-associated changes in DNA methylation using label-free SERS. Specifically, we demonstrate that Ca^2+^ —along with Mg² ⁺ , Be² ⁺ , Zn² ⁺ , and Al³ ⁺ — ions facilitate the adsorption of DNA onto silver nanoparticles and activate the DNA SERS signal. Additionally, we, identify the SERS band at 790 cm^-1^ as a marker of DNA methylation, and demonstrate that hypermethylated DNA exhibits a stronger affinity for silver surfaces.

## Experimental

### Genomic DNA purification

Genomic DNA (gDNA) was extracted from the following immortalized human cell lines: the benign cell lines HaCaT (immortalized keratinocytes) and LX-2 (human hepatic stellate cells), as well as the malignant cell lines SK-HEP-1 (hepatocellular carcinoma), MDA-MB-231 (triple-negative breast cancer), A549 (lung cancer), and HCT116 (colon cancer) (Table 1). Both non-transformed and malignant cell lines were included to control for cancer-specific epigenetic alterations. For gDNA extraction, cell suspensions containing no more than 5 million cells in PBS (Gibco) were prepared. To each suspension, 20 µL of Proteinase K and 20 µL of RNase A (both from Promega) were added, followed by vortexing and incubation at room temperature for 2 minutes. Subsequently, 200 µL of lysis/binding buffer (PureLink Genomic DNA Mini Kit, Thermo Fisher Scientific, K182002) were added and mixed by vortexing for 5 seconds. Samples were then incubated at 55°C for 10 minutes to promote digestion. Following incubation, 200 µL of molecular-grade ethanol was added to the mixture, which was then vortexed to ensure thorough homogenization. A 640 µL aliquot of the mixture was loaded onto a PureLink Spin column fitted with a collection tube and centrifuged at 10,000 × g for 1 minute at room temperature. After discarding the collection tube, 500 µL of Wash Buffer 1 (prepared with ethanol) was added, and the column was centrifuged again at 10,000 × g for 1 minute. The process was repeated with 500 µL of Wash Buffer 2, followed by centrifugation at 17,850 × g for 2 minutes. After discarding the collection tube, a final centrifugation step was performed at 17,850 × g for 3 minutes to remove residual ethanol. The spin column was then placed into a sterile, DNAse/RNAse-free 1.5 mL microtube, and gDNA was eluted using 50 µL of ultrapure nuclease-free water instead of the standard elution buffer. This substitution was critical, as the standard elution buffer contains EDTA, which chelates calcium ions (Ca^2+^) essential for DNA adsorption onto silver nanoparticles (AgNPs) during SERS analysis. Eluting with ultrapure water preserved Ca^2+^ availability, enabling successful acquisition of SERS spectra, whereas DNA eluted with standard buffer failed to produce detectable SERS signals (S1 Fig and S2 Fig). The concentration and purity of extracted gDNA were assessed using a Nanodrop spectrophotometer (Thermo Fisher Scientific), ensuring that the A260/A280 ratio exceeded 1.8 and the A260/A230 ratio exceeded 2.0 for all samples. Extracted DNA was stored at –80°C until further use.

**Table 1:** Overview of DNA samples analysed in this study.

DNA sample	Type	Source
Calf thymus DNA	Genomic DNA, double-stranded, size ≤2,000 bp	Sigma
Genomic DNA from cell lines HaCaT, LX-2 SK-HEP-1, MDA-MB-231, A549, and HCT116	Genomic DNA, double stranded, size distribution not available.	Extracted by PureLink Genomic DNA Mini Kit, Thermo Fisher Scientific, #K182002
*SEPT9*	Short sequence, double-straned, size = 180 bp (three methylation levels)	GenScript

### Additional DNA sources and methylation analysis

In addition to gDNA from cell lines, commercially available calf thymus gDNA (Sigma) and a chemically synthesized 180 bp DNA fragment corresponding to the SEPT9 gene promoter (GenScript) were also used. Three methylation states were analyszed in the case of the SEPT9 sequences: fully unmethylated (control), 50% methylated (specific methylated cytosines marked in bold below), and fully methylated. The global cytosine methylation levels of gDNA were quantified independently for each DNA extraction batch using a Global DNA Methylation Assay Kit (ELISA-based, Abcam). All chemicals and reagents used in this study were of analytical grade.

The sequence of the SEPT9 gene promoter was the following:

5’-CTCCTCGAGGGCTCGCGAGGCTGCCTCGGAACTCTCCAGGACGCACAGTTTCACTCTGGGAAATCCATCGGTCCCCTCCCTTTGGCTCTCCCCGGCGGCTCTCGGGCCCCGCTTGGACCCGGCAACGGGATAGGGAGGTCGTTCCTCACCTCCGACTGAGTGGACAGCCGCGTCCTGCTC-3’

### Nanoparticle synthesis

Colloidal AgNPs were synthesized by reducing Ag⁺ ions with hydroxylamine hydrochloride (hya-AgNPs), following a previously established method [36]. Briefly, 17 mg of AgNO_3_ was dissolved in 90 mL of ultrapure water under constant stirring. Separately, 17 mg of hydroxylamine hydrochloride was dissolved in 8.8 mL of ultrapure water, and 1.2 mL of 1% NaOH was added. The hydroxylamine solution was then introduced into the AgNO_3_ solution under stirring, resulting in an immediate color change to brown, indicating nanoparticle formation. The colloidal suspension was stored at ambient conditions until use.

To investigate the role of cations in DNA adsorption onto silver surfaces, we also prepared citrate-reduced AgNPs (cit-AgNPs) [37]. In this procedure, 17 mg of AgNO₃ was dissolved in 98 mL of ultrapure water and heated to boiling while stirring continuously. Subsequently, 2 mL of 1% sodium citrate solution was added dropwise. The mixture was continuously boiled and stirred for 30 minutes to complete nanoparticle formation.

### Surface-enhanced Raman scattering (SERS)

To control for the DNA concentration as a confounding variable, all DNA samples were diluted to a uniform concentration of 20 ng/µL (unless otherwise stated). For SERS measurements, 5 µL of AgNPs was mixed with 5 µL of DNA solution and 1 µL of Ca(NO₃)₂ (final Ca^2+^ concentration of 5x10^-4^ M). A 5 µL aliquot of the mixture was deposited onto a microscope slide covered with aluminium foil for spectral acquisition.

SERS spectra were acquired using an InVia Raman spectrometer (Renishaw) equipped with a frequency-doubled Nd:YAG laser (532 nm, approximately 50 mW output), focused through a 5X objective lens (Leica, NA 0.12). Each spectrum was averaged from three successive acquisitions of 10 seconds each. Measurements were conducted in liquid drop form. The reproducibility of the SERS spectra for gDNA was validated using DNA extracted from NB4 and HaCaT cell lines, by recording six spectra within two hours (S3 Fig).

### Fluorescence measurements

Fluorescence measurements were conducted to quantify unbound gDNA after incubation with hya-AgNPs. The technique is based on dyes that become fluorescent when bound to double-stranded DNA. However, the adsorption of DNA (and probably of the dye) onto the nanoparticles leads to the quenching of the fluorescent signal. For fluorescent measurements, 140 ng of gDNA was mixed with 1 mL of hya-AgNPs at concentrations ranging from 0.3 x10^10^ to 4.75x10^10^ nanoparticles/mL, corresponding to silver surface areas between 8 and 134 mm^2^ (see the details for this calculation in the next paragraph). To promote DNA adsorption, Ca(NO_3_)_2_ was added to a final concentration of 5x10^-4^ M. After incubation, samples were centrifuged at 7300 x g for 15 minutes, and the supernatant was collected for analysis. For fluorescence labelling, 180 µL of the supernatant was mixed with 20 µL of SYBR Green (Invitrogen), diluted 1:10,000 in ultrapure water, and transferred into a 96-well plate. Fluorescence emission was measured using a microplate reader (Thermo Fisher Scientific), which enabled quantification of free, unbound gDNA remaining in solution. Samples were excited at 470 ± 20 nm, and fluorescence emission was collected at 520 nm. Measurements were performed in triplicate, and the mean and standard deviation were calculated. The proportion of DNA bound to nanoparticles relative to the total available surface area was modelled using the Langmuir adsorption isotherm, as implemented in GraphPad Prism 6 (GraphPad Software Inc.). The model provides estimates for two key parameters: the maximum binding capacity (B_max_) and the equilibrium dissociation constant (K_d_). B_max_ represents the theoretical saturation point of binding sites, while K_d_ indicates the analyte concentration required to achieve half of B_max_, reflecting the binding affinity, with lower K_d_ values corresponding to stronger binding interactions.

To monitor DNA adsorption as a function of available silver surface area, the concentration of hya-AgNPs was varied. Nanoparticle concentration was determined using nanoparticle tracking analysis (Nanosight NS300, Malvern), and particle size was measured using dynamic light scattering (Zetasizer, Malvern). Assuming spherical geometry (mean diameter: 60 ± 6 nm), the surface area of a single hya-AgNP was calculated to be approximately 2826 nm². The total available surface area was obtained by multiplying the nanoparticle number by the estimated surface area per particle.

## Results and discussion

### The role of atomic cations in turning on the SERS spectrum of DNA

**Fig 1:**
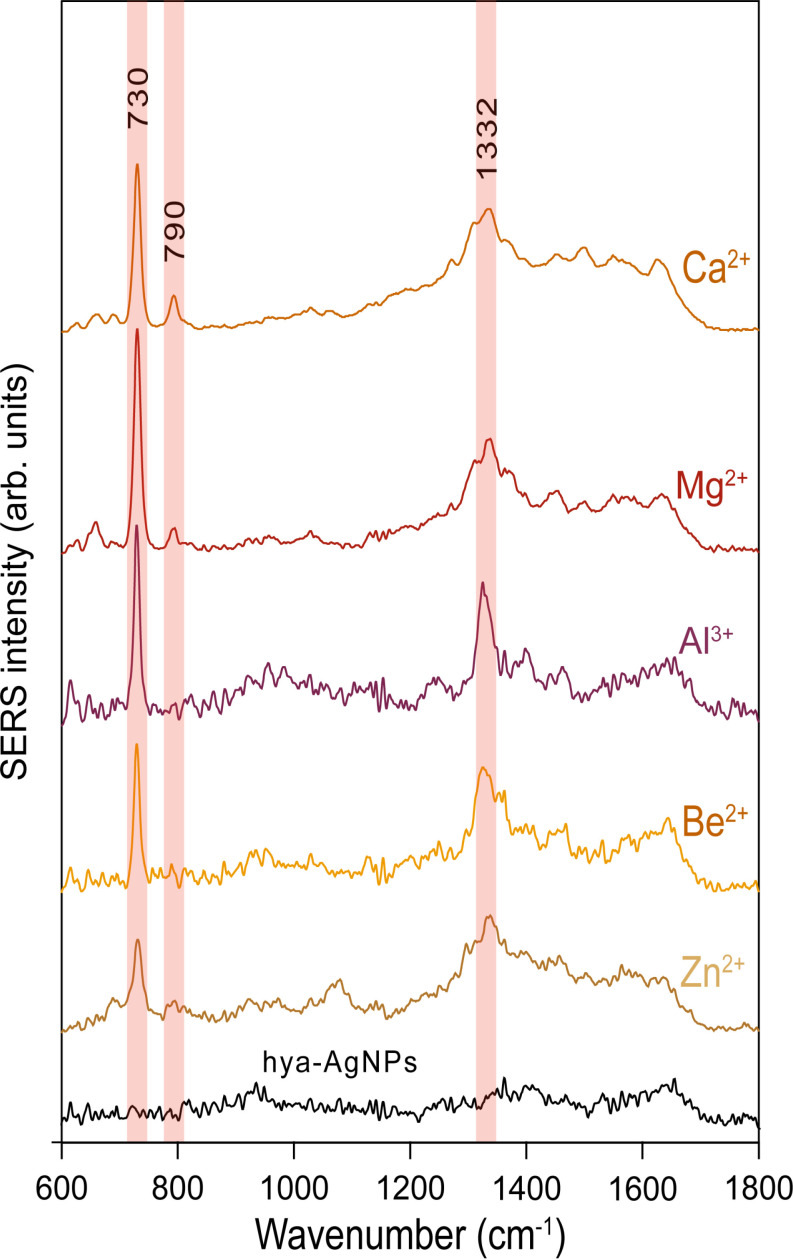
DNA adsorption promoted by cations and subsequent SERS detection. SERS spectra of calf thymus DNA (20 ng/µL) acquired after the addition of 5x10^-4^ M ZnSO_4_, BeSO_4_, Ca(NO_3_)_2_, MgSO_4_, Al_2_(SO_4_)_3_. The SERS spectrum of DNA with the SERS substrate (hya-AgNPs), without any added cations, was blank (black spectrum).

As with many other analytes, simply mixing DNA with a substrate does not yield a SERS spectrum. We have previously demonstrated that cations such Ag^+^, Ca^2+^ and Al^3+^ turn on the SERS spectrum of negatively charged organic acids [20], while Mg^2+^ turns on the SERS spectrum of the anionic dye Rose Bengal [19]. Using a similar approach, we evaluated the ability of several cations at a concentration of 5 × 10 ⁻⁴ M to activate the SERS signal of calf thymus genomic DNA, employing hya-AgNPs as SERS substrate. The results showed that Ca^2+^, Mg^2+^, Be^2+^, Zn^2+^ and Al^3+^ are all able to activate the SERS signal of gDNA, as evidenced by typical SERS bands of adenine at 730, 1330 cm^-1^ and cytosine and 5-methylcytosine at 790 cm^-1^ [38–41] (Fig 1).

Among the cations, the highest signal-to-noise ratio was achieved in the presence of highly hydrated cations such as Ca^2+^ and Mg^2+^. In contrast, we found that atomic cations such as Cu^2+^, Fe^2+^, and Na^+^ ions do not turn on the SERS signal of DNA (S4 Fig). This finding suggests that the adsorption effect is not solely dependent on electrostatic interactions, since Cu^2+^ and Fe^2+^ possess the same charge as Ca^2+^ and Mg^2+^. Instead, the results suggest that the turning on of the SERS signal of DNA is triggered by the adsorption of the analyte. Several cations have been previously reported to activate the SERS signal of DNA, including MgSO_4_ [17,23]_,_ Zr(NO_3_)_4_ [9] or combinations [42,43]. However, it is important to note that these studies did not provide detailed information about nanoparticle aggregation or the adsorption behavior of DNA. As a result, the exact mechanism of signal enhancement should be interpreted with caution. To better clarify the role of these cations, future studies should include a systematic evaluation of analyte adsorption and nanoparticle aggregation under well-controlled conditions.

### The role of Ca^2^^+^ for promoting the adsorption of DNA onto silver nanoparticles (AgNPs)

As previously discussed in our paper [18], a hallmark of SERS detection is the ability to selectively adsorb specific analytes within a mixture. To demonstrate this principle using calf thymus gDNA, we conducted sequential SERS and fluorescence measurements, beginning with a mixture of hya-AgNPs and gDNA, followed by the stepwise addition of Ca²⁺ and subsequently iodide (I ⁻) . SERS and fluorescence assays using DNA-binding dyes such as SYBR Green offer complementary information: SERS detects signals exclusively from molecules adsorbed onto the metal surface, while unbound molecules remain undetected. Conversely, fluorescence measurements using SYBR Green enable the quantification of unbound DNA (see the Experimental section for additional details regarding SYBR Green methodology).

**Fig 2:**
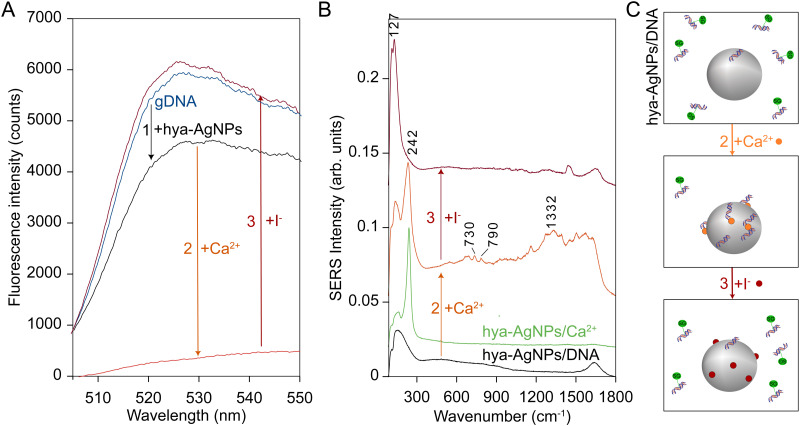
The adsorption dynamics of genomic DNA (gDNA) on silver nanoparticles (hya-AgNPs) monitored by SERS and fluorescence. (A) Fluorescence emission of SYBR Green-labeled gDNA (blue) slightly decreases upon adding hya-AgNPs (black, arrow 1), indicating partial adsorption. Adding 5x10^-4^ M Ca² ⁺ caused a sharp fluorescence drop (orange, arrow 2), signifying extensive gDNA adsorption. Subsequent addition of 10^−3^ M I⁻ restored fluorescence (red, arrow 3), indicating complete desorption. (B) SERS spectra of AgNPs with gDNA showed initially no signal (black). The addition of Ca^2+^ turned on the SERS spectrum gDNA (orange, arrow 2). The addition of Ca^2+^ also turned on a SERS band at 242 cm^-1^ attributed to Ag-Cl (chloride is already present in the hya-AgNPs from the synthesis process). Adding I⁻  led to the dissapearance of the SERS spectrum of DNA and the appearance of a SERS band at 127 cm^-1^ attributed to Ag-I (red, arrow 3). Adding Ca^2+^ on hya-AgNPs lead to the appearance of the SERS band at 242 cm^-1^ even in the absence of gDNA (green spectrum). (C) A schematic illustration of the adsorption processes presented in panel A, B (green dot- SYBR Green, orange dot-Ca^2+^, red dot-I^-^).

The results of this experiment confirmed that the activation of the DNA SERS signal by Ca^2+^ is indeed due to DNA adsorption (Fig 2). Thus, when gDNA labeled with SYBR Green was mixed with colloidal hya-AgNPs, only a slight decrease in fluorescence was observed (Fig 2A, blue and black spectra), and the corresponding SERS spectrum displayed only the Raman band of water (Fig 2B, black spectrum), indicating that the DNA remained largely unbound. Following the addition of Ca^2+^, a substantial decrease in fluorescence was detected, signifying the adsorption of gDNA onto the AgNP surfaces (Fig 2A, red spectrum). Simultaneously, the SERS signal of DNA was activated (Fig 2B, red spectrum), evidenced by the appearance of characteristic bands corresponding to cytosine (790 cm^-1^), adenine (730 and 1330 cm^-1^), and guanine (680 cm^-1^). In addition, Ca^2+^ supplementation led to the adsorption of Cl⁻ ions (present from the synthesis process), as indicated by the emergence of a SERS band at 242 cm^-1^ (Fig 2B, red spectrum). Notably, this Cl⁻ band at 242 cm^-1^ was also observed when hya-AgNPs were mixed with Ca^2+^ in the absence of DNA (Fig 2B, green spectrum).

While most protocols for obtaining label-free SERS spectra of DNA rely on nanoparticle aggregation induced by salts such as MgSO_4_ [17,23,26] our approach is primarily based on promoting DNA adsorption onto the nanoparticle surface rather than inducing aggregation. To confirm that the observed SERS signal of DNA results from reversible adsorption rather than irreversible aggregation, we introduced I⁻ ions, which have a much higher affinity for silver surfaces than gDNA. As expected, the addition of I ⁻ led to the reappearance of strong SYBR Green fluorescence (Fig 2A, magenta spectrum), indicating gDNA displacement by I^-^, accompanied by the disappearance of DNA-specific SERS bands, due to desorption from the surface, and the emergence of a new SERS band at 127 cm^-1^, corresponding to the formation of an I-Ag bond (Fig 2B, magenta spectrum). These results suggest that Ca^2+^ effectively promotes the selective chemisorption of analytes, with an induced affinity series as follows: I⁻ > gDNA ≥ Cl ⁻ .

To demonstrate the broader applicability of this effect, we investigated a different system comprising citrate-capped silver nanoparticles (cit-AgNPs), DNA, and Cl⁻ ions (S5 Fig). The results indicated that the relative affinity for silver surfaces in the presence of Ca^2+^ follows the order: DNA ≥ Cl⁻ > citrate. Thus, upon adding Ca^2+^ to mixtures of gDNA and cit-AgNPs, characteristic DNA SERS bands were observed at 730 and 1332 cm^-1^ (assigned to adenine) and at 790 cm^-1^ (assigned to cytosine) (S5 Fig). However, residual SERS bands corresponding to citrate were also detected (marked with asterisks in S5 Fig). To eliminate interference from citrate, Cl⁻ ions were added to the solution, successfully displacing citrate from the nanoparticle surface, as evidenced by the disappearance of citrate-associated SERS bands while preserving the DNA-specific signals. Additionally, the Ag–Cl vibrational band appeared at 242 cm^-1^, which did not interfere with the SERS spectrum of DNA (S5 Fig). These findings underscore the importance of selecting a surfactant that does not produce overlapping SERS signals within the spectral region of DNA. A comparable “cleaning” effect, involving the removal of artifact bands in DNA sensing, was previously reported with cit-AgNPs treated with KI in the case of short DNA fragments [26].

It has been previously suggested that Ca^2+^ binds to the phosphate groups (PO_4_^2-^) in DNA, thereby decreasing the electrostatic repulsion between the DNA backbone and the negatively charged surfactant on the nanoparticle [44]. If this hypothesis were correct, then DNA fragments at significantly lower concentrations (1 ng/µL) should require much less Ca^2+^ for becoming adsorbed onto AgNPs compared to genomic DNA at 20 ng/µL. However, this hypothesis was contradicted by the experimental findings, which showed no detectable SERS signal for the DNA fragments at 5x10^-4^ M Ca^2+^; instead, a significantly higher concentration (up to 5x10^-2^ M) was required to observe a SERS signal (S6 Fig).

Another hypothesis put forward was that the role of Ca^2+^ has to do with surfactant complexation [44], thus counteracting the electrostatic repulsion. However, this hypothesis is incompatible with the experimental findings, which showed that Ca^2+^ can turn on the SERS spectrum of DNA irrespective of the SERS surfactant (citrate for cit-AgNPs and Cl^-^ for hya-AgNPs). Collectively, these findings indicate that the role of Ca² ⁺ is more likely linked to modification of the metal surface, rather than to neutralization of analyte charge or surfactant complexation, as further demonstrated in the following paragraph.

We observed a similar SERS turn-on effect for Ca²⁺ as in the case of lowering the pH of the solution (S7 Fig). A slight decrease in the pH of the colloidal solution is known to initiate the dissolution of surface silver, leading to the formation of Ag⁺ sites on the silver surface [20]. We propose that Ca² ⁺ may induce a similar effect. These newly formed Ag⁺ adatoms could then interact directly with DNA bases, in agreement with previous reports [45–47]. Supporting this interpretation, we successfully obtained SERS signals from calf thymus gDNA by lowering the solution pH instead of adding Ca^2+^ (S7 Fig), consistent with the known ability of acidic conditions to induce substrate dissolution via metal oxidation [48]. Based on these findings, we propose that the role of Ca^2+^ is primarily related to modifying the surface charge of the AgNPs rather than simply influencing the electrostatic properties of DNA. Using low salt concentrations, which prevent nanoparticle aggregation, results in improved reproducibility of SERS signals compared to aggregated substrates, where signal variability is typically higher due to inconsistent hotspot formation.

### Correlation between the intensity of the cytosine SERS band at 790 cm^-1^ and DNA methylation levels

**Fig 3:**
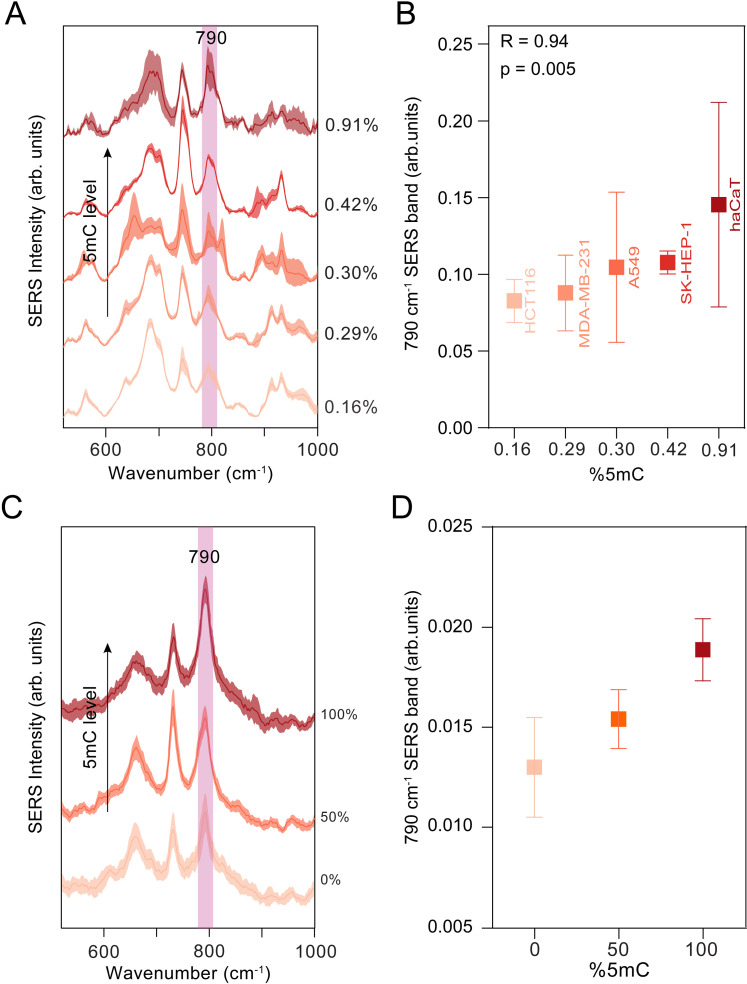
Correlation between the intensity of the cytosine SERS band at 790 cm^-1^ and DNA methylation levels. (A) SERS spectra presented as mean and standard deviation of six genomic DNA (gDNA) samples (10 ng/µL) with 5-methylcytosine (5mC) levels ranging from 0.16 to 0.91%. The silver colloidal solution was supplemented with 5x10^-4^ M Ca^2+^. (B) Positive correlation between intensity of cytosine SERS band at 790 cm^-1^ and the levels of 5mC. Boxes represent average; whiskers represent standard deviation (C) The SERS spectra (mean and standard deviation) of a double-stranded DNA sequence of 180 bp from the promoter region of *SEPT9* gene with 0%, 50% and 100% methylation levels. (D) The correlation between the levels of 5mC and the intensity of the SERS band at 790 cm^-1^. Boxes represent average; whiskers represent standard deviation.

To demonstrate the potential of label-free SERS for assessing DNA methylation levels, we aimed to identify the most suitable SERS marker band for this purpose. We extracted gDNA from five cell lines, which exhibited varying methylation levels ranging from 0.16% to 0.91% (with the exception of HaCaT, all were malignant cell lines), and recorded their SERS spectra using hya-AgNPs supplemented with 5x10^-4^ M Ca^2+^ (Fig 3A; a broader spectral region is shown in S8 Fig). Upon analysing the SERS spectra of DNA, we identified the cytosine band at 790 cm ⁻ ¹ as the most reliable marker, with its intensity showing a strong correlation with DNA methylation levels (Pearson correlation coefficient R = 0.94, p = 0.005; Fig 3B).

The strong positive correlation between the cytosine SERS band at 790 cm^-1^ and the levels of 5-methylcytosine in gDNA extracted from different cell lines underscores the potential of SERS to quantify 5-methylcytosine concentration in DNA.

To demonstrate the potential for measuring DNA methylation in a clinically relevant context, we analysed a 180 bp sequence from the promoter region of the human *SEPT9* gene [49], the first FDA-approved methylation marker for colorectal cancer screening (see the Experimental section for the full DNA sequence) [50]. Comparative SERS spectra of fully unmethylated, partially methylated, and fully methylated S*EPT9* DNA revealed a stepwise increase in the intensity of the SERS band at 790 cm ⁻ ¹ (Fig 3C, D). For the fully methylated *SEPT9* sequence, intense SERS bands could be discerned for concentrations as low as 0.05 ng/µL (S9 Fig).

The label-free quantification of DNA methylation has been previously reported on short single-stranded and double-stranded DNA sequences using zirconium-modified AgNPs [9] or on genomic DNA in which methylation levels were altered through enzymatic treatment [10]. The authors identified several different SERS marker bands for DNA methylation; however, these findings should be interpreted in light of the experimental differences (short DNA sequences or enzymatic methylation). We have also previously reported that DNA methylation is linked to a SERS band near 1008 cm^-1^ [51]. However, this band appears to be more susceptible to batch effects and occasionally displays low intensity, thus highlighting the challenges in identifying reliable marker bands for biologically significant covalent modifications of DNA.

### Hypermethylated DNA has an increased affinity for silver surfaces

**Fig 4:**
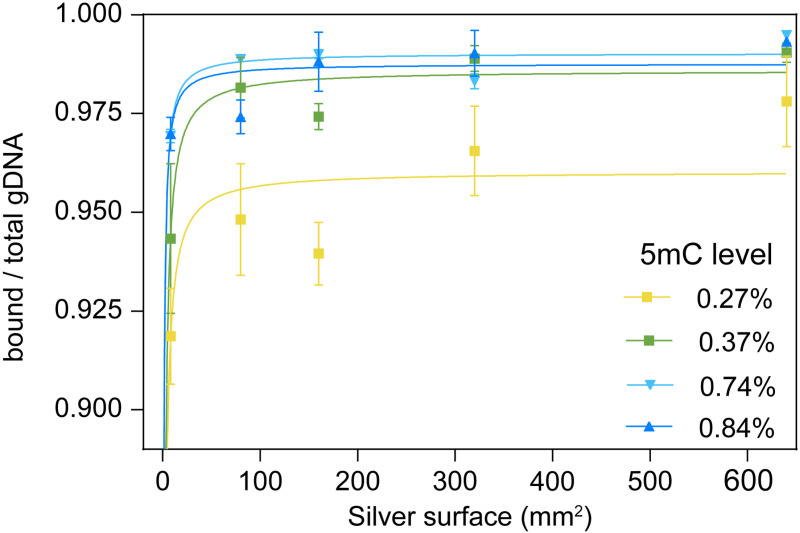
The effect of DNA methylation on the affinity for silver surfaces. Adsorption rate of genomic DNA (gDNA) for various levels of 5-methylcytosine (5mC) in relation to the total silver surface.

To assess whether DNA methylation influences the affinity of DNA for the silver surface, we measured the proportion of bound DNA across different concentrations of hya-AgNPs, effectively varying the total available silver surface area. The proportion of DNA bound to the nanoparticles was determined by comparing the fluorescence emission intensity of SYBR Green between the initial amount of gDNA (100 ng) and the remaining unbound gDNA in the supernatant. This value was normalized to the total DNA amount, plotted against the total available silver surface (see Experimental section for calculation details), and analysed using the Langmuir adsorption model. The results demonstrated that hypermethylated gDNA exhibited an increased adsorption affinity for the silver surface (Fig 4). Moreover, a strong negative correlation was observed between DNA methylation levels and the equilibrium dissociation constant (K_d_) (Table 2; Pearson correlation coefficient R = –0.993, p = 0.006).

**Table 2:** The Langmuir fit parameters for the adsorption rate of gDNA to the silver surface. K_d_ is the equilibrium dissociation constant. B_max_ represents the maximum binding capacity. Methylation levels are indicated in %.

	A549 (0.27%)	HCT116 (0.37%)	SK-HEP-1 (0.74%)	LX-2 (0.84%)
B_max_	0.960	0.986	0.990	0.99
K_d_	0.38	0.36	0.17	0.15

The observed negative correlation between DNA methylation levels and the equilibrium dissociation constant (K_d_) indicates that hypermethylated DNA exhibits stronger affinity for silver surfaces, a finding with important implications for the selective biosensing of cancer-derived DNA. This effect has been reported before [35]. However, the underlying mechanism responsible for the differential binding affinity based on methylation status remains unclear. Increased methylation has been linked to greater hydrophobicity and molecular rigidity [52], which may contribute to an enhanced affinity for metal surfaces. Nonetheless, additional research is required to fully understand this behaviour.

The observation that hypermethylated DNA exhibits a stronger affinity for silver surfaces is particularly significant in clinical contexts, where the allelic frequency of hypermethylated DNA markers such as *SEPT9* is typically very low. Therefore, this enhanced affinity may offer the critical specificity required for effective application in real-world clinical diagnostics.

SERS offers several advantages over conventional analytical techniques for DNA methylation analysis [53] such as whole genome bisulfite sequencing [54], ELISA [55] or LC-MS [56]. The primary advantages are the exceptionally fast turnaround time—typically within a few minutes—low cost, and non-destructive nature of the method, which allows the DNA to be recovered intact after analysis. In addition, SERS generally requires less input DNA compared to whole-genome bisulfite sequencing, which typically demands hundreds of nanograms to several micrograms, and ELISA or LC-MS methods, which often require tens of nanograms. Although newer versions of these techniques have aimed to lower input requirements, SERS remains highly efficient in this regard. The amount of DNA needed for SERS depends on the DNA type—ranging from as little as 0.5 ng for short oligonucleotides (S9 Fig) to several tens of nanograms for genomic DNA.

It is important to note that one source of variability not addressed in this study stems from the use of (bulk) genomic DNA, which presents significant heterogeneity in its epigenetic landscape. While unsupervised clustering of the SERS spectra suggests additional spectral variations that may reflect gene-specific methylation changes (S10 Fig), further investigation is required to establish SERS’s capacity for precise, gene-specific methylation profiling. Differences in chromatin organization (euchromatin versus heterochromatin) [57], fragmentation patterns [58], the presence of repetitive DNA elements [59], and other epigenetic modifications beyond methylation—such as acetylation and hydromethylation [60]—may all contribute to variability in binding behavior.

## Conclusions

In this study, we report three key findings regarding the adsorption of DNA onto silver surfaces. Firstly, we demonstrated that DNA adsorption onto AgNPs occurs only in the presence of certain cations, specifically Ca^2+^, Mg^2+^, Be^2+^, Zn^2+^, and Al^3+^. Among these, highly hydrated ions such as Ca^2+^ and Mg^2+^ produced the most favorable signal-to-noise ratios in the SERS spectra of DNA. In contrast, the addition of Cu^2+^, Fe^2+^, or Na ⁺ did not facilitate DNA adsorption onto AgNPs. Furthermore, we provided evidence suggesting that the SERS activation effect of Ca^2+^ is mediated by its ability to generate positive metal ions (Ag⁺) on the SERS substrate, a phenomenon that can also be induced by lowering the pH. Maintaining low salt concentrations to prevent nanoparticle aggregation enhances the reproducibility of SERS signals, which is essential for ensuring reliability—particularly in clinical applications where consistency and accuracy are critical for diagnostic use. 

Secondly, we established a significant positive correlation between the intensity of the DNA SERS band at 790 cm^-1^ and the level of 5-methylcytosine, highlighting the potential of label-free SERS for monitoring DNA methylation. We further validated this approach by analysing a 180 bp region from the promoter of the *SEPT9* gene, one of the few FDA-approved methylation markers for colorectal cancer, and demonstrated the ability of SERS to distinguish hypermethylation. Future studies are needed to evaluate whether SERS can detect hypermethylation in clinical samples where the proportion of hypermethylated DNA is substantially lower.

Finally, we demonstrated that hypermethylated DNA binds more strongly to silver surfaces, providing the key specificity needed for practical use in clinical diagnostic settings.

SERS provides a rapid, cost-effective, and non-destructive alternative to conventional DNA methylation analysis methods such as whole-genome bisulfite sequencing, ELISA, and LC-MS, while requiring considerably less DNA input—highlighting its strong potential for sensitive, scalable, and clinically relevant diagnostic applications.

Collectively, these findings have important theoretical and practical implications for the development of future nanoparticle-based platforms for DNA methylation detection and quantification.

## Supporting information

S1 Fig The SERS spectra of the components of the DNA extraction kit. The experimental parameters were similar to those used for DNA SERS detection (see the main manuscript). The DNA was extracted using the PureLink Genomic DNA Mini Kit, Thermo Fisher Scientific. (DOCX)

S2 Fig The lack of SERS amplification when DNA was eluted with elution buffer. The EDTA-containing elution buffer from the PureLink Genomic DNA Mini Kit, Thermo Fisher Scientific was used. (DOCX)

S3 Fig Reproducibility of SERS spectra of genomic DNA. DNA was extracted from non-transformed human immortalized keratinocytes cells (HaCaT) and malignant human acute promyelocytic leukemia cells (NB4). The depicted spectra represent the average from six different extractions. The spectra were acquired within two hours from mixing 5 µL of AgNPs with 5 µL of DNA (20 ng/µL) and 1 µL of Ca(NO_3_)_2_ (final concentration 5x10^-4^ M Ca^2+^). Each individual spectrum represents the average of three acquisitions, 10 second each. Shaded areas represent the standard deviation. (DOCX)

S4 Fig Lack of SERS enhancement for DNA in the presence of Na ⁺ , Fe² ⁺ , Cu² ⁺ . The SERS spectra of 100 ng calf thymus DNA (10 ng/mL) obtained by mixing 5 µL of DNA with 5 µL of silver nanoparticles synthesized by reductio with hydroxylamine hydrochloride (hya-AgNPs) and 5x10^-4^ M Na_2_SO_4_, FeSO_4_, CuSO_4_. (DOCX)

S5 Fig The adsorption of calf thymus DNA onto citrate-capped silver nanoparticles in the presence of Ca^2+^ ions. From bottom to top: the initial SERS spectrum of cit-AgNPs with Ca²⁺ (Ca(NO_3_)_2_ at 5 x 10^−4^ M) is shown in black, displaying characteristic citrate bands. Upon the addition of ctDNA, DNA-specific SERS bands emerged (blue), although several citrate-related artifact bands (indicated with *) remained visible. Subsequent addition of Cl⁻ ions resulted in citrate desorption from the silver surface, allowing clear observation of the DNA SERS spectrum (purple). cit-AgNPs = citrate-capped silver nanoparticles; ctDNA = calf thymus DNA. (DOCX)

S6 Fig The influence of Ca^2+^ (Ca(NO_3_)_2_) concentration on the SERS spectra of DNA fragments. The concentration of DNA was 1 ng/µL. The full sequence of the primers is shown in the main article. (DOCX)

S7 Fig Effect of acidic pH on the SERS spectra of genomic DNA. The DNA was extracted from the NB4 cell line (1 ng/μL). The green spectrum represents the SERS signal of a mixture of genomic DNA and hydroxylamine-reduced silver nanoparticles (hya-AgNPs) at neutral pH, while the orange spectrum shows the same solution under acidic conditions (pH 4). (DOCX)

S8 Fig The full SERS spectra of genomic DNA extracted from cell lines. SERS spectra presented as mean and standard deviation of six genomic DNA samples (20 ng/µl) with 5-methylcytosine (5mC) concentrations ranging from 0.16 to 0.91%. (DOCX)

S9 Fig The SERS spectra of a 180 bp double-stranded DNA sequence from the promoter region of the SEPT9 gene. The amount of DNA was then analysed in 10 µL of silver nanoparticles synthesized by reduction with hydroxylamine hydrochloride supplemented with Ca(NO_3_)_2_ 5x10^-4^ M. See the Experimental section of the main manuscript for the sequence of the fragment. (DOCX)

S10 Fig The results of the t-SNE Unsupervised clustering of the SERS spectra of genomic DNA from cell lines. The t-SNE was calculated on the first 20 Principal Components on the SERS spectra of genomic from HaCaT (immortalized keratinocytes) and LX-2 (human hepatic stellate cell), and the malignant cell lines SK-HEP-1 (hepatocellular carcinoma), MDA-MB-231 (triple-negative breast cancer), A549 (lung cancer), HCT116 (colon cancer). The t-SNE was calculated using the Quasar software (Orange). (DOCX)
