# Supplementary material for: SERS-based detection of DNA methylation for cancer diagnosis: Cation-mediated adsorption to silver nanoparticles
Source: PLoS One. 2025 Jun 13;20(6):e0325539. doi: 10.1371/journal.pone.0325539 (PMC12165392; doi:10.1371/journal.pone.0325539)
Supplement: S1 Fig — (DOCX) [file pone.0325539.s001.docx]

**
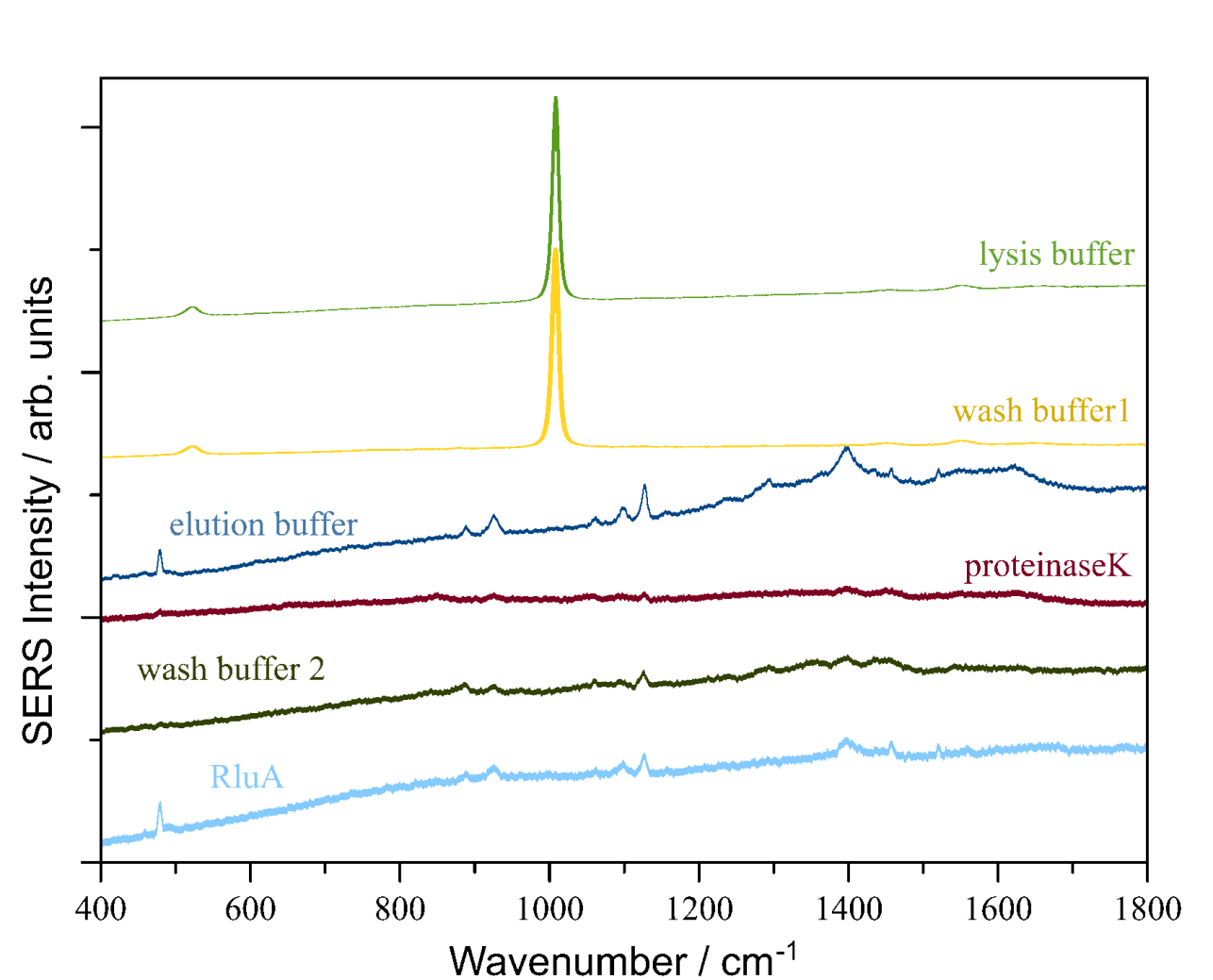
**

**Supplementary Figure 1.** The SERS spectra of the components of the DNA extraction kit. The experimental parameters were similar to those used for DNA SERS detection (see the main manuscript). The DNA was extracted using the PureLink Genomic DNA Mini Kit, Thermo Fisher Scientific.
