# Supplementary material for: SERS-based detection of DNA methylation for cancer diagnosis: Cation-mediated adsorption to silver nanoparticles
Source: PLoS One. 2025 Jun 13;20(6):e0325539. doi: 10.1371/journal.pone.0325539 (PMC12165392; doi:10.1371/journal.pone.0325539)
Supplement: S2 Fig — (DOCX) [file pone.0325539.s002.docx]

**
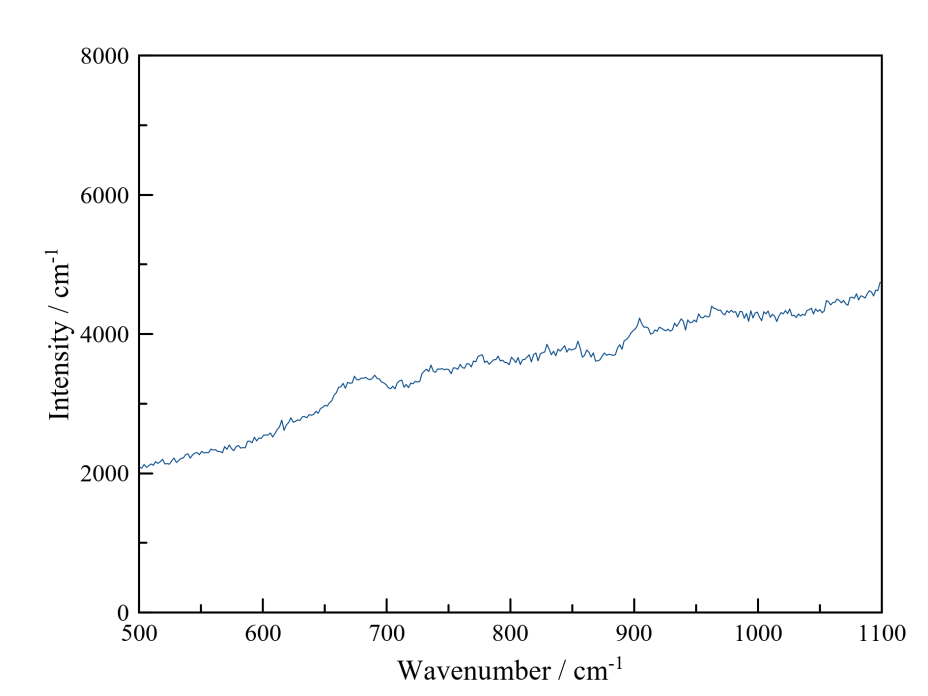
**

**Supplementary Figure 2.** The lack of SERS amplification when DNA was eluted with elution buffer. The EDTA-containing elution buffer from the PureLink Genomic DNA Mini Kit, Thermo Fisher Scientific.
