# Supplementary material for: SERS-based detection of DNA methylation for cancer diagnosis: Cation-mediated adsorption to silver nanoparticles
Source: PLoS One. 2025 Jun 13;20(6):e0325539. doi: 10.1371/journal.pone.0325539 (PMC12165392; doi:10.1371/journal.pone.0325539)
Supplement: S3 Fig — (DOCX) [file pone.0325539.s003.docx]

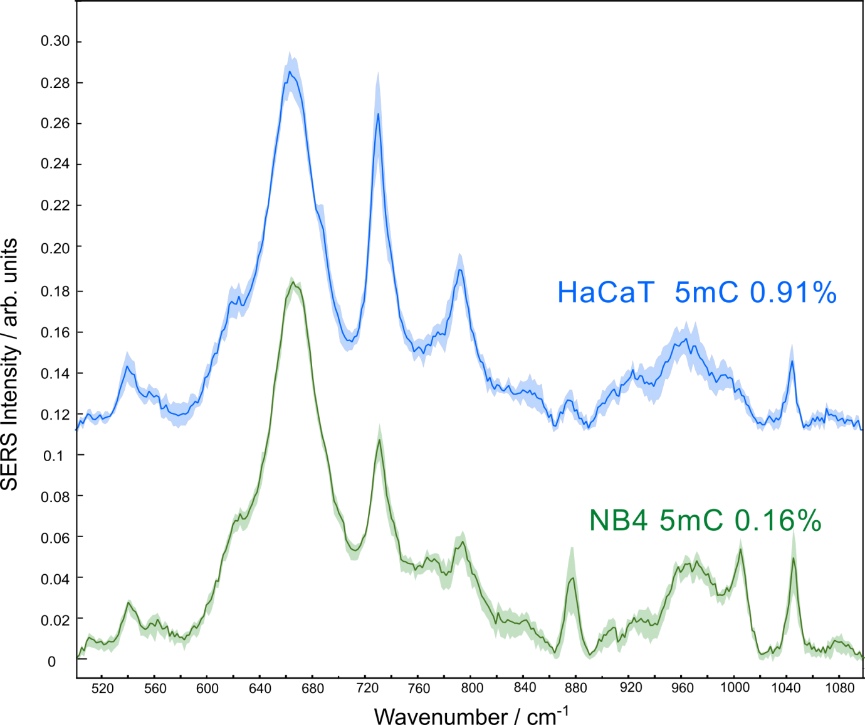


**Supplementary Figure 3**. Reproducibility of SERS spectra of genomic DNA. DNA was extracted from non-transformed human immortalized keratinocytes cells (HaCaT) and malignant human acute promyelocytic leukemia cells (NB4). The depicted spectra represent the average from six different extractions. The spectra were acquired within two hours from mixing 5 µL of AgNPs with 5 µL of DNA (20 ng/µL) and 1 µL of Ca(NO_3_)_2_ (final concentration 5x10^-4^ M Ca^2+^). Each individual spectrum represents the average of three acquisitions, 10 second each. Shaded areas represent the standard deviation.
