# Supplementary material for: SERS-based detection of DNA methylation for cancer diagnosis: Cation-mediated adsorption to silver nanoparticles
Source: PLoS One. 2025 Jun 13;20(6):e0325539. doi: 10.1371/journal.pone.0325539 (PMC12165392; doi:10.1371/journal.pone.0325539)
Supplement: S4 Fig — (DOCX) [file pone.0325539.s004.docx]

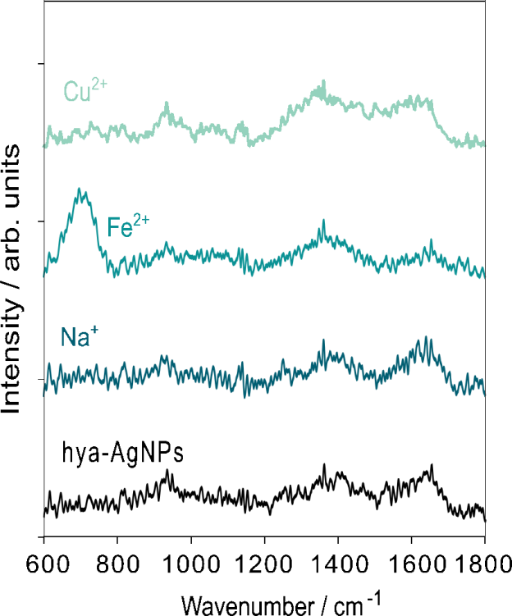


**Supplementary Figure 4.** Lack of SERS enhancement for DNA in the presence of Na⁺, Fe²⁺, Cu²⁺. The SERS spectra of 100 ng calf thymus DNA (20 ng/mL) obtained by mixing 5 µL of DNA with 5µL of silver nanoparticles synthesized by reductio with hydroxylamine hydrochloride (hya-AgNPs) and 5x10^-4^ M Na_2_SO_4_, FeSO_4_, CuSO_4_.
