# Supplementary material for: SERS-based detection of DNA methylation for cancer diagnosis: Cation-mediated adsorption to silver nanoparticles
Source: PLoS One. 2025 Jun 13;20(6):e0325539. doi: 10.1371/journal.pone.0325539 (PMC12165392; doi:10.1371/journal.pone.0325539)
Supplement: S5 Fig — (DOCX) [file pone.0325539.s005.docx]

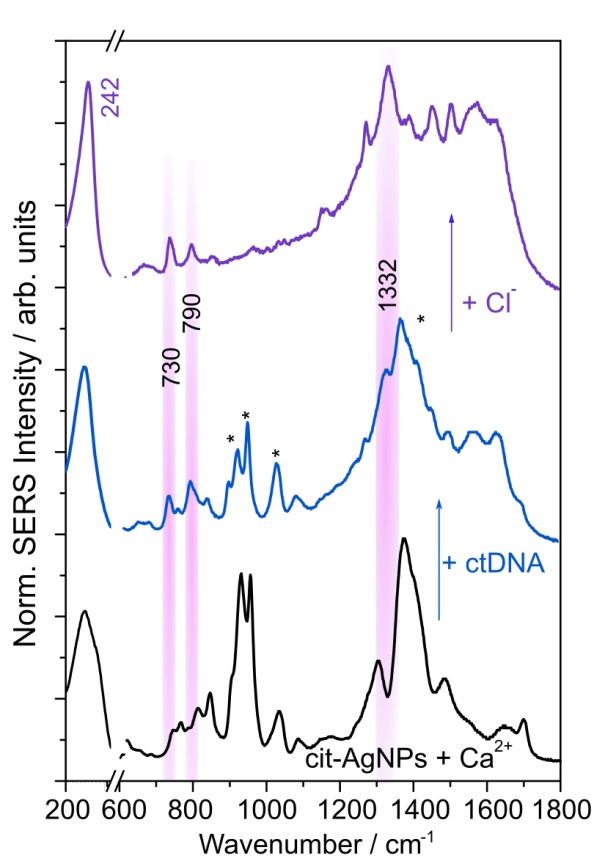


**Supplementary Figure 5.** The adsorption of calf thymus DNA onto citrate-capped silver nanoparticles in the presence of Ca^2+^ ions. From bottom to top: the initial SERS spectrum of cit-AgNPs with Ca²⁺ (Ca(NO_3_)_2_ at 5*10^-4^ M) is shown in black, displaying characteristic citrate bands. Upon the addition of ctDNA, DNA-specific SERS bands emerged (blue), although several citrate-related artifact bands (indicated with *) remained visible. Subsequent addition of Cl⁻ ions resulted in citrate desorption from the silver surface, allowing clear observation of the DNA SERS spectrum (purple). cit-AgNPs = citrate-capped silver nanoparticles; ctDNA = calf thymus DNA.
