# Supplementary material for: SERS-based detection of DNA methylation for cancer diagnosis: Cation-mediated adsorption to silver nanoparticles
Source: PLoS One. 2025 Jun 13;20(6):e0325539. doi: 10.1371/journal.pone.0325539 (PMC12165392; doi:10.1371/journal.pone.0325539)
Supplement: S6 Fig — (DOCX) [file pone.0325539.s006.docx]

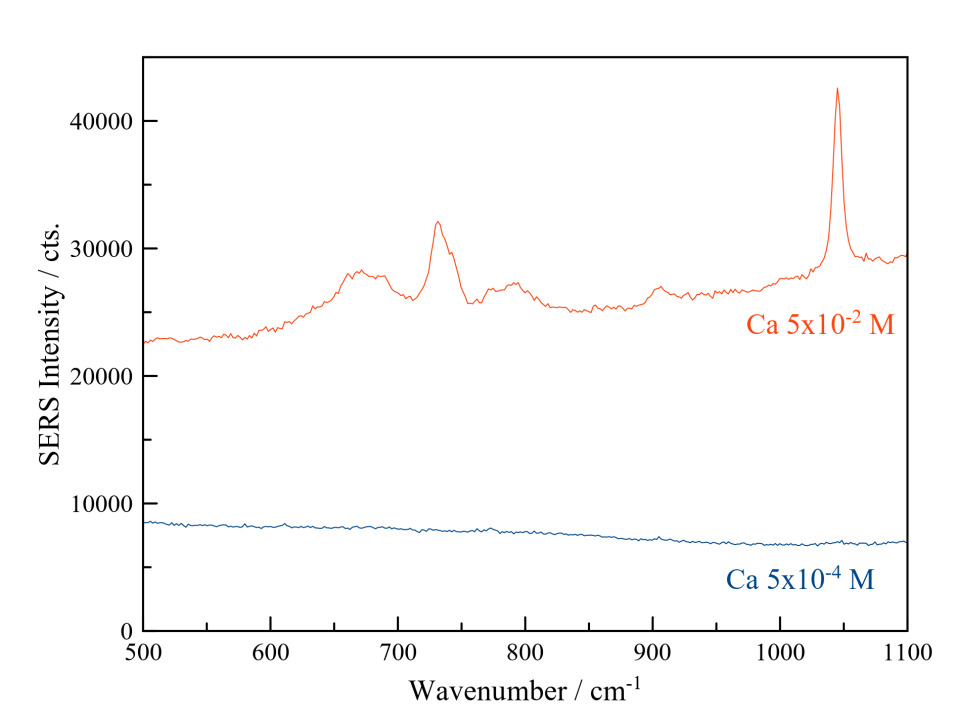


**Supplementary Figure 6.** The influence of Ca^2+^ (Ca(NO_3_)_2_) concentration on the SERS spectra of DNA fragments. The concentration of DNA was 1 ng/µL. The full sequence of the primers is shown in the main article.
