# Supplementary material for: SERS-based detection of DNA methylation for cancer diagnosis: Cation-mediated adsorption to silver nanoparticles
Source: PLoS One. 2025 Jun 13;20(6):e0325539. doi: 10.1371/journal.pone.0325539 (PMC12165392; doi:10.1371/journal.pone.0325539)
Supplement: S7 Fig — (DOCX) [file pone.0325539.s007.docx]

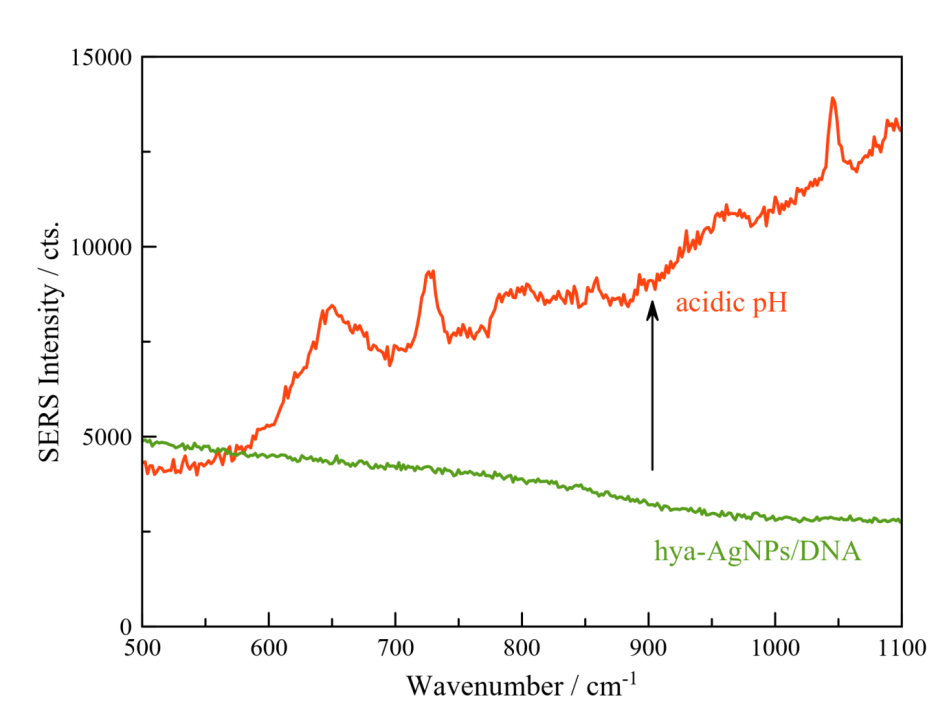


**Supplementary Figure 7.** Effect of acidic pH on the SERS spectra of genomic DNA. The DNA was extracted from the NB4 cell line (1 ng/μL). The green spectrum represents the SERS signal of a mixture of genomic DNA and hydroxylamine-reduced silver nanoparticles (hya-AgNPs) at neutral pH, while the orange spectrum shows the same solution under acidic conditions (pH 4).
