# Supplementary material for: SERS-based detection of DNA methylation for cancer diagnosis: Cation-mediated adsorption to silver nanoparticles
Source: PLoS One. 2025 Jun 13;20(6):e0325539. doi: 10.1371/journal.pone.0325539 (PMC12165392; doi:10.1371/journal.pone.0325539)
Supplement: S8 Fig — (DOCX) [file pone.0325539.s008.docx]

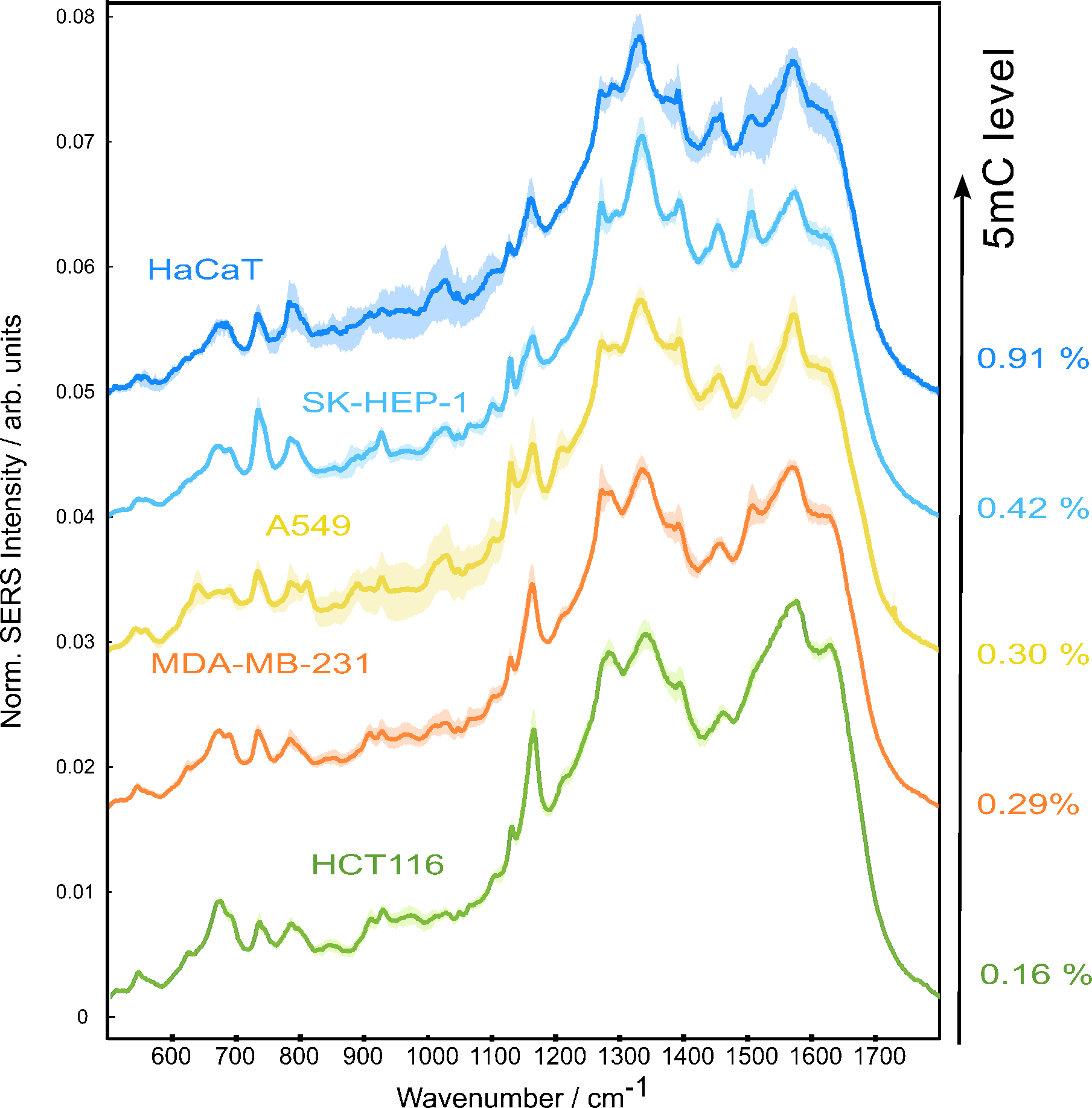


**Supplementary** **Figure 8.** The full SERS spectra of genomic DNA extracted from cell lines. SERS spectra presented as mean and standard deviation of six genomic DNA samples (20 ng/µl) with 5-methylcytosine (5mC) concentrations ranging from 0.16 to 0.91%.
