# Supplementary material for: SERS-based detection of DNA methylation for cancer diagnosis: Cation-mediated adsorption to silver nanoparticles
Source: PLoS One. 2025 Jun 13;20(6):e0325539. doi: 10.1371/journal.pone.0325539 (PMC12165392; doi:10.1371/journal.pone.0325539)
Supplement: S9 Fig — (DOCX) [file pone.0325539.s009.docx]

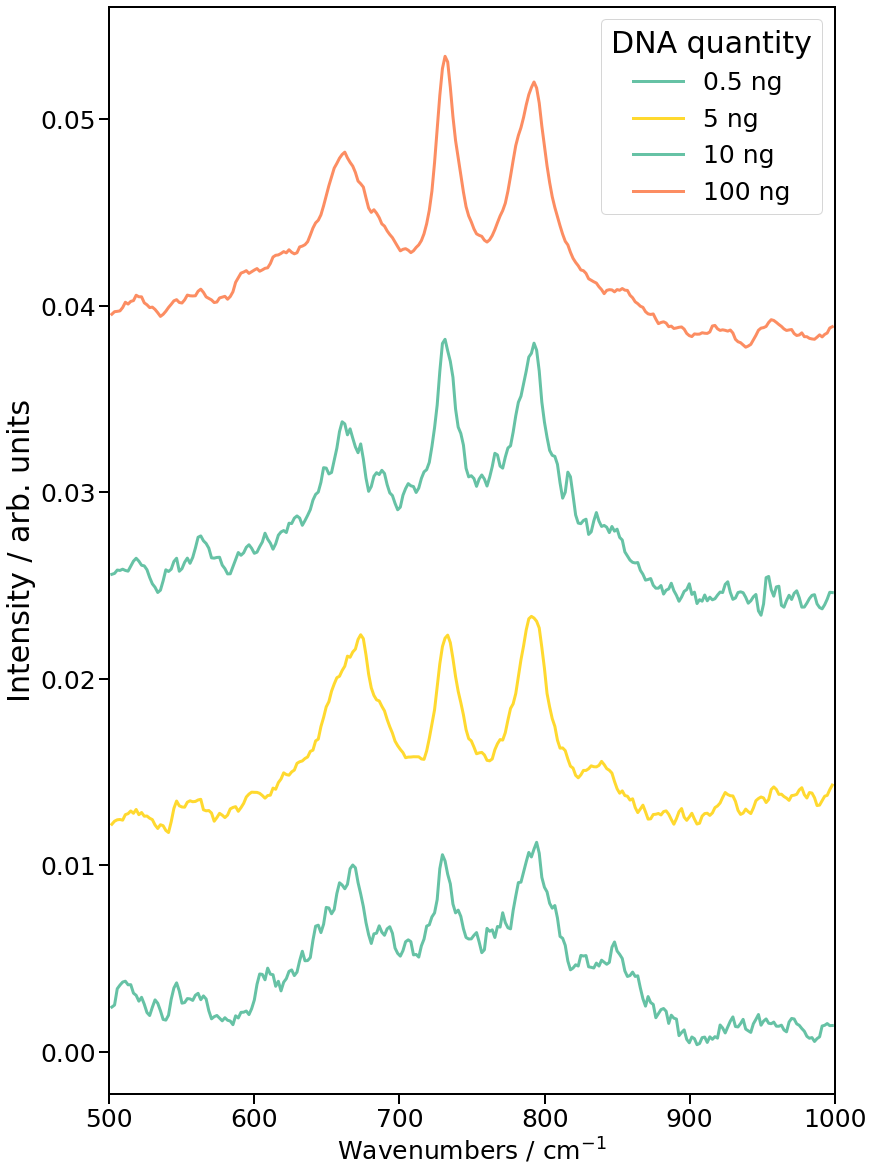


**Supplementary Figure 9.** The SERS spectra of a 180 bp double-stranded DNA sequence from the promoter region of the SEPT9 gene. The amount of DNA was then analyzed in 10 µL of silver nanoparticles synthesized by reduction with hydroxylamine hydrochloride supplemented with Ca(NO_3_)_2_ 5x10^-4^ M. See the Experimental section of the main manuscript for the sequence of the fragment.
