# Supplementary material for: SERS-based detection of DNA methylation for cancer diagnosis: Cation-mediated adsorption to silver nanoparticles
Source: PLoS One. 2025 Jun 13;20(6):e0325539. doi: 10.1371/journal.pone.0325539 (PMC12165392; doi:10.1371/journal.pone.0325539)
Supplement: S10 Fig — (DOCX) [file pone.0325539.s010.docx]

**
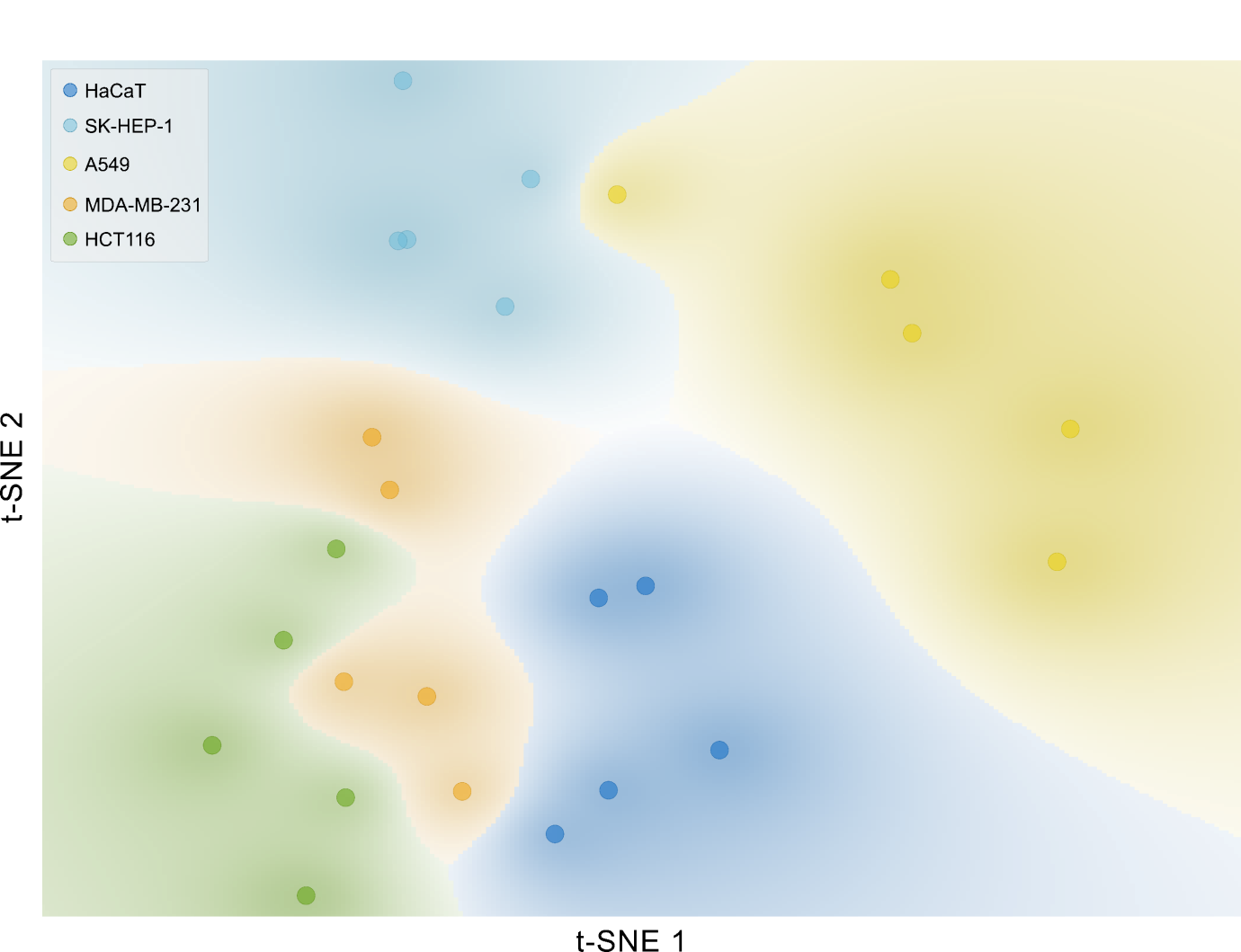
**

**Supplementary Figure 10.** The results of the t-SNE Unsupervised clustering of the SERS spectra of genomic DNA from cell lines. The t-SNE was calculated on the first 20 Principal Components on the SERS spectra of genomic from HaCaT (immortalized keratinocytes) and LX-2 (human hepatic stellate cell), and the malignant cell lines SK-HEP-1 (hepatocellular carcinoma), MDA-MB-231 (breast cancer), A549 (lung cancer), HCT116 (colon cancer). The t-SNE was calculated using the Quasar software (Orange).
